# Factors associated with poor cardiometabolic control in patients with type 2 diabetes: a Lasso-Firth penalized regression analysis

**DOI:** 10.3389/fendo.2026.1905392

**Published:** 2026-09-03

**Authors:** Wei Yang, Da-shuai Xie, Chao Song, Jing Li, Fei-fei Wu, Xiao Zhang, Le Ma, Yao-jun Ning, Jian-feng Wang, Rong-hong Li, Li-hong Hao, Pei Gu, Ping Shi, Xiao-jing Wang, Qin Zhang

**Affiliations:** 1 Department of Pharmacy, Yuncheng Central Hospital Affiliated to Shanxi Medical University, Yuncheng, China; 2 Department of Endocrinology, Shanxi Province Hospital of Traditional Chinese Medicine, Taiyuan, China; 3 Department of Endocrinology, Jincheng People’s Hospital, Jincheng, China; 4 Department of Endocrinology, Heping Hospital, Changzhi Medical College, Changzhi, China; 5 Department of Endocrinology, Linfen People’s Hospital, Linfen, China; 6 Department of Pharmacy, Yangquan Coal Industry (Group) General Hospital, Yangquan, China; 7 Department of Endocrinology, Datong Tongmei Hospital, Datong, China; 8 Department of Endocrinology, Yuncheng Central Hospital Affiliated to Shanxi Medical University, Yuncheng, China; 9 Department of Orthopedics, Yuncheng Central Hospital Affiliated to Shanxi Medical University, Yuncheng, China

**Keywords:** blood glucose, blood lipids, blood pressure, diabetic, KAP, Lasso-Firth penalized regression analysis

## Abstract

**Objective:**

To assess cardiometabolic control among patients with type 2 diabetes mellitus (T2DM) in Shanxi Province and identify variables associated with poor cardiometabolic control.

**Methods:**

Data on demographic characteristics, medical history, Knowledge–Attitude–Practice (KAP) questionnaire scores, and clinical measurements of blood pressure, glucose, and lipid levels were collected from seven hospitals in Shanxi Province between June 2024 and June 2025. Poor cardiometabolic control was defined as failure to achieve comprehensive control across the three cardiometabolic domains (blood pressure, glycemia, and lipid levels), with failure to meet the target in any single domain considered poor control. Variable selection was performed using the least absolute shrinkage and selection operator (LASSO) combined with bootstrap-based stability selection. Firth’s penalized logistic regression was then applied to the stably selected variables to estimate their associations with poor cardiometabolic control.

**Results:**

Among 1,874 patients, only 2.88% achieved comprehensive cardiometabolic control. Most patients had marked deficits in knowledge of blood pressure and lipid management, as well as in self-monitoring and symptom recognition. Bootstrap stability selection combined with Firth regression identified six candidate variables. Among these, BMI, education level, and knowledge score exhibited the strongest, nominally significant associations with poor cardiometabolic control, whereas practice score, hypertension history, and T2DM duration were selected but did not reach nominal significance. These exploratory findings require validation in independent cohorts.

**Conclusions:**

Cardiometabolic control among patients with T2DM in Shanxi Province remains suboptimal. Exploratory analyses suggest that BMI, education level, and knowledge score are the variables most robustly associated with poor cardiometabolic control, whereas practice score, hypertension history, and T2DM duration represent additional, weaker candidate associations warranting further investigation. These findings may inform future diabetes self-management education and intervention strategies aimed at improving glycemic, blood pressure, and lipid control. However, given the cross-sectional and exploratory nature of this study, the results should be regarded as hypothesis-generating and require confirmation in prospective or interventional studies before causal inferences can be drawn.

## 1 Introduction

Type 2 diabetes mellitus (T2DM) is a chronic metabolic disorder characterized by insulin resistance or impaired insulin secretion (1, 2). Persistent hyperglycemia causes progressive systemic tissue damage and contributes to multiple organ complications, posing a major global public health challenge (3). According to the International Diabetes Federation, approximately 537 million people worldwide were living with diabetes worldwide in 2021, with related healthcare expenditures reaching US$966 billion; these costs are projected to exceed US$1,054 billion by 2045 (4). T2DM frequently coexists with cardiovascular risk factors, particularly hypertension and hyperlipidemia. These interrelated conditions share common pathophysiological mechanisms that accelerate atherosclerosis and promote target-organ damage affecting the heart, brain, and kidneys, thereby increasing diabetes-related morbidity and mortality (5).

The WHO Global Diabetes Coverage Targets for 2030 and the Healthy China Initiative (2019–2030) identify standardized management of blood glucose, blood pressure, and lipid levels as key objectives of diabetes prevention and control (6, 7). Nevertheless, substantial gaps remain between these recommendations and their implementation in clinical practice (8). Worldwide, relatively few patients achieve simultaneous control of all three cardiometabolic risk factors (9–11). For example, only 2.9% of patients with T2DM in Shanghai, China, achieved all three treatment targets (12). Despite the clinical importance of comprehensive cardiometabolic management, relatively few studies have systematically investigated variables associated with simultaneous glycemic, blood pressure, and lipid control in patients with T2DM.

With global population aging, the prevalence of obesity and diabetes continues to rise, driving a concurrent increase in cardiovascular morbidity and mortality. Consequently, it is essential to assess control of blood pressure, glycemia, and lipids and to identify variables associated with inadequate control in patients with T2DM. To this end, the Knowledge-Attitude-Practice (KAP) model is a well-established behavioral framework proposing that health-related knowledge shapes attitudes, which in turn influence self-care practice, ultimately affecting health outcomes (13, 14). This model has been widely applied in health education, chronic disease management, and evaluation of public health interventions (12, 15). Within the context of type 2 diabetes, the framework suggests that disease-related knowledge, psychosocial attitudes toward self-management, and self-care practice are interrelated factors potentially associated with glycemic, lipid, and blood pressure control. Accordingly, the KAP model was used to investigate diabetes-related knowledge, attitudes, and self-care practice among patients across seven hospitals in Shanxi Province, China. We then examined the associations of demographic characteristics, medical history, and KAP information with poor cardiometabolic control, applying LASSO combined with bootstrap-based stability selection and Firth’s penalized logistic regression to identify candidate variables associated with this outcome.

## 2 Methods

### 2.1 Patient population

This cross-sectional survey was conducted in Shanxi Province, China, using a stratified random sampling approach. Seven cities were randomly selected from 11 administrative regions of Shanxi. One hospital was then chosen from each selected city, resulting in seven geographically distributed sites from north to south: Datong Tongmei Hospital; Yangquan Coal Industry (Group) General Hospital; Shanxi Provincial Hospital of Traditional Chinese Medicine; Jincheng People’s Hospital; Linfen People’s Hospital; Heping Hospital affiliated with Changzhi Medical College; and Yuncheng Central Hospital. The survey was conducted at these sites between June 2024 and June 2025. The study protocol was reviewed and approved by the Ethics Committee of Yuncheng Central Hospital, affiliated to Shanxi Medical University (Approval No. YXLL2024055). Written informed consent was obtained from all participants before questionnaire administration and data collection.

### 2.2 Data collection

Data collection comprised two components: questionnaire data and clinical laboratory data. Questionnaire data were primarily collected using Wenjuanxing, a professional online survey platform with built-in field validation and logical consistency checks to minimize missing data. Participants who preferred not to use the online platform completed paper-based questionnaires, which were subsequently entered into the platform by trained researchers and manually cross-checked to minimize transcription errors. For elderly participants or those with limited literacy who had difficulty completing the questionnaire independently, trained endocrinologists, nurses, or clinical pharmacists provided face-to-face assistance, guiding these participants through the questionnaire items and resolving queries on site to maintain data accuracy and consistency.

Demographic characteristics included age, gender, body mass index (BMI), monthly income, place of residence, health insurance coverage, education level, working conditions, marital status, and smoking status. Medical history comprised family history of diabetes mellitus (FHDM), duration of T2DM (T2DM duration), history of hypertension (HHTN), history of stroke (Stroke history), history of coronary heart disease (CHD), acute coronary syndrome within the past year (ACS), symptomatic peripheral vascular disease (PVDWS), family history of premature coronary heart disease (FHCHD), familial hypercholesterolemia (FH), prior coronary artery bypass grafting or percutaneous coronary intervention (CABG or PCI), and renal insufficiency (RI).

Clinical and laboratory data were extracted directly from the Electronic Medical Record (EMR) systems by the information technology (IT) departments of the seven participating hospitals, thereby minimizing the risk of manual transcription errors. Because all participating hospitals are located within Shanxi Province and participate in the regional clinical laboratory mutual recognition system, laboratory test results are generated under standardized quality-control procedures and are considered comparable across institutions. Blood pressure, glycemic, and lipid control were assessed using systolic and diastolic blood pressure, fasting plasma glucose (FPG), glycated hemoglobin (HbA1c), total cholesterol (TC), triglycerides (TG), high-density lipoprotein cholesterol (HDL-C), and low-density lipoprotein cholesterol (LDL-C). Target values (**Table 1**) were defined according to the Chinese Guidelines for the Prevention and Treatment of Type 2 Diabetes (2020 edition) and were used to determine whether participants achieved the recommended treatment goals. A composite outcome representing comprehensive cardiometabolic control was defined as simultaneous achievement of the target levels for blood pressure, glycemia, and lipid control. Participants who failed to achieve one or more of these targets were classified as having poor cardiometabolic control. The composite outcome was defined in accordance with the comprehensive cardiometabolic control targets specified in the WHO Global Diabetes Coverage Targets (16) and the Healthy China Initiative (17), which explicitly define adequate diabetes management as the simultaneous control of blood glucose, blood pressure, and lipids, reflecting the clinical reality that residual risk from any single uncontrolled component continues to drive atherosclerotic and end-organ complications (18).

**Table 1:** Targets for integrated control of blood glucose, blood pressure, and lipids in type 2 diabetes.

Indices	Target value
Blood glucose (mmol/L)	
Fasting	4.4-7.0
Non-fasting	<10.0
HbA1c	<7.0
Blood pressure (mmHg)	<130/80
TC (mmol/L)	<4.5
HDL-C (mmol/L)	
male	>1.0
female	>1.3
TG (mmol/L)	<1.7
LDL-C (mmol/L)	
ASCVD not present	<2.6
ASCVD	<1.8
BMI (kg/m^2^)	<24.0

### 2.3 Knowledge-attitude-practice evaluation

The revised Chinese version of the Diabetes, Hypertension, and Hyperlipidemia Knowledge Scale (DHL) was used to assess disease-related knowledge (12, 19). The scale consists of 28 items covering five domains: diabetes, hypertension, hyperlipidemia, medication, and general knowledge. Each item was answered as “true,” “false,” or “don’t know.” Correct responses were assigned 1 point, whereas incorrect or “don’t know” responses received 0 points. Total scores were converted to a standardized percentage scale (0–100), with higher scores indicating greater disease-related knowledge.

Attitudes toward diabetes were assessed using the Chinese version of the Diabetes Empowerment Scale–Short Form (DES-SF) (12), which consists of eight items. Responses were rated on a 5-point Likert scale ranging from 1 (strongly disagree) to 5 (strongly agree). Item scores were summed and converted to a standardized percentage scale (0–100), with higher scores indicating more positive attitudes toward diabetes self-management.

Self-care practice was assessed using the Chinese version of the Self-Care of Diabetes Inventory (SCODI) (20). The SCODI comprises 40 items across four domains: self-care maintenance, self-care monitoring, self-care management, and self-care confidence. Previous studies have suggested that self-care confidence is associated with behavior change and may facilitate self-care performance. Therefore, this domain was retained as part of the behavioral assessment framework in the present study (17). All items were rated on a 5-point Likert scale. For the maintenance, monitoring, and management domains, response options ranged from 1 (never) to 5 (always), whereas those for the confidence domain ranged from 1 (not at all confident) to 5 (very confident). Total scores were converted to a standardized percentage scale (0–100), with higher scores indicating better self-care practice.

### 2.4 Statistical analysis

The two datasets were subsequently merged using unique participant identifiers, with identifiers stored as text strings to prevent automatic data conversion or removal of leading zeros. Final data cleaning, variable reconciliation, and statistical analyses were performed exclusively using R software (version 4.5.1). Normally distributed continuous variables were summarized as means ± standard deviations (SDs) and compared using Student’s t-test. Non-normally distributed continuous variables were summarized as medians with interquartile ranges (IQRs) and compared using the Mann–Whitney U test. Categorical variables were presented as numbers (percentages) and compared using the chi-square test.

Canonical correlation analysis (CCA) was used to examine relationships among demographic characteristics (X1), medical history(X2), KAP scores (X3), as independent variable sets, and cardiometabolic control (Y) as the dependent variable set. The first canonical variate pair represents the maximum possible correlation between any linear combinations of variables in X and Y, respectively; accordingly, we focused on the first canonical correlation (21). Structure coefficients were visualized using canonical correlation helio plots.

For the CCA, cardiometabolic control was represented by its three constituent components (attainment of blood pressure, glycemic, and lipid targets) to characterize how demographic, medical history, and KAP variables relate to the pattern of control across these three domains. By contrast, in the subsequent LASSO–Firth regression analysis, cardiometabolic control was operationalized as a single composite binary outcome, defined as the simultaneous attainment of all three treatment targets, consistent with this study’s definition of comprehensive cardiometabolic control.

In this study, this single composite binary outcome served as the dependent variable, and demographic characteristics, medical history, and KAP score served as independent variables in the LASSO-Firth model. During data collection, we observed a high proportion of “unknown” responses regarding specialized cardiovascular medical histories, such as ACS and FH, likely attributable to limited health literacy and the technical nature of the terminology involved. In clinical epidemiological surveys, an “unknown” response to such major ischemic events or genetic disorders is generally interpreted as the absence of a confirmed diagnosis. To reduce multicollinearity and overfitting in the multivariable models, and consistent with internationally recognized Atherosclerotic Cardiovascular Disease (ASCVD) risk stratification guidelines, nine variables were aggregated into two composite variables: Stroke history, CHD, ACS, PVDWS, CABG, and PCI were combined to define a single ASCVD variable, and FHDM, FHCHD, and FH were combined into a family history of cardiovascular disease (FHCVD) variable. Each composite variable was coded as 1 (yes) if the patient responded “yes” to any constituent item, and 0 (no/unconfirmed) if all responses were “no” or “unknown.” This aggregation was applied only for the LASSO-Firth analysis, to reduce collinearity and overfitting given the limited number of outcome events.

The LASSO regression was used to perform initial variable selection and coefficient shrinkage, thereby identifying candidate variables of poor cardiometabolic control while minimizing the risk of overfitting. Variance inflation factors (VIFs) were calculated before LASSO selection and after the final set of stable variables had been determined, to assess multicollinearity; a VIF < 5 was considered indicative of the absence of substantial multicollinearity. To improve the robustness of variable selection beyond a single LASSO fit, bootstrap-based stability selection (1,000 resamples) was performed (22), and variables with a selection frequency of ≥ 75% were retained as stable variables. A selection-frequency threshold of 75% was prespecified to define stably selected variables. This threshold falls within the range commonly used in the stability-selection literature and corresponded to a natural gap in the observed selection-frequency distribution. Sensitivity analyses using alternative thresholds (60%–80%) were performed to evaluate the robustness of variable selection.

Because of the low outcome event rate, approaches requiring data splitting or asymptotically valid post-selection inference were not considered feasible. Instead, bootstrap-based stability selection was used to improve the robustness of variable selection before Firth penalized logistic regression (23, 24). The stably selected variables were subsequently entered into a Firth penalized logistic regression model, and the results were summarized as odds ratios (ORs), 95% confidence intervals (CIs), and *P* values. Because the regression model was fitted to the same dataset used for variable selection, post-selection uncertainty was not formally accounted for. Accordingly, the resulting ORs, CIs, and *P* values were interpreted as exploratory and hypothesis-generating rather than confirmatory.

To further characterize the association between knowledge score and poor cardiometabolic control and to evaluate potential dose-response relationships, knowledge score was stratified into quartiles (Q1–Q4) as a pre-specified sensitivity analysis, and Firth’s logistic regression was used to calculate ORs and 95% CIs with Q1 as the reference category. Linear trends (*P* for trend) were assessed by modeling the quartiles as a continuous ordinal variable. A subgroup analysis was performed to evaluate whether the association between knowledge score and the outcome varied across population subgroups. Statistical significance was defined as *P*<0.05.

## 3 Result

### 3.1 Basic information and medical history of participants

**Figure 1:**
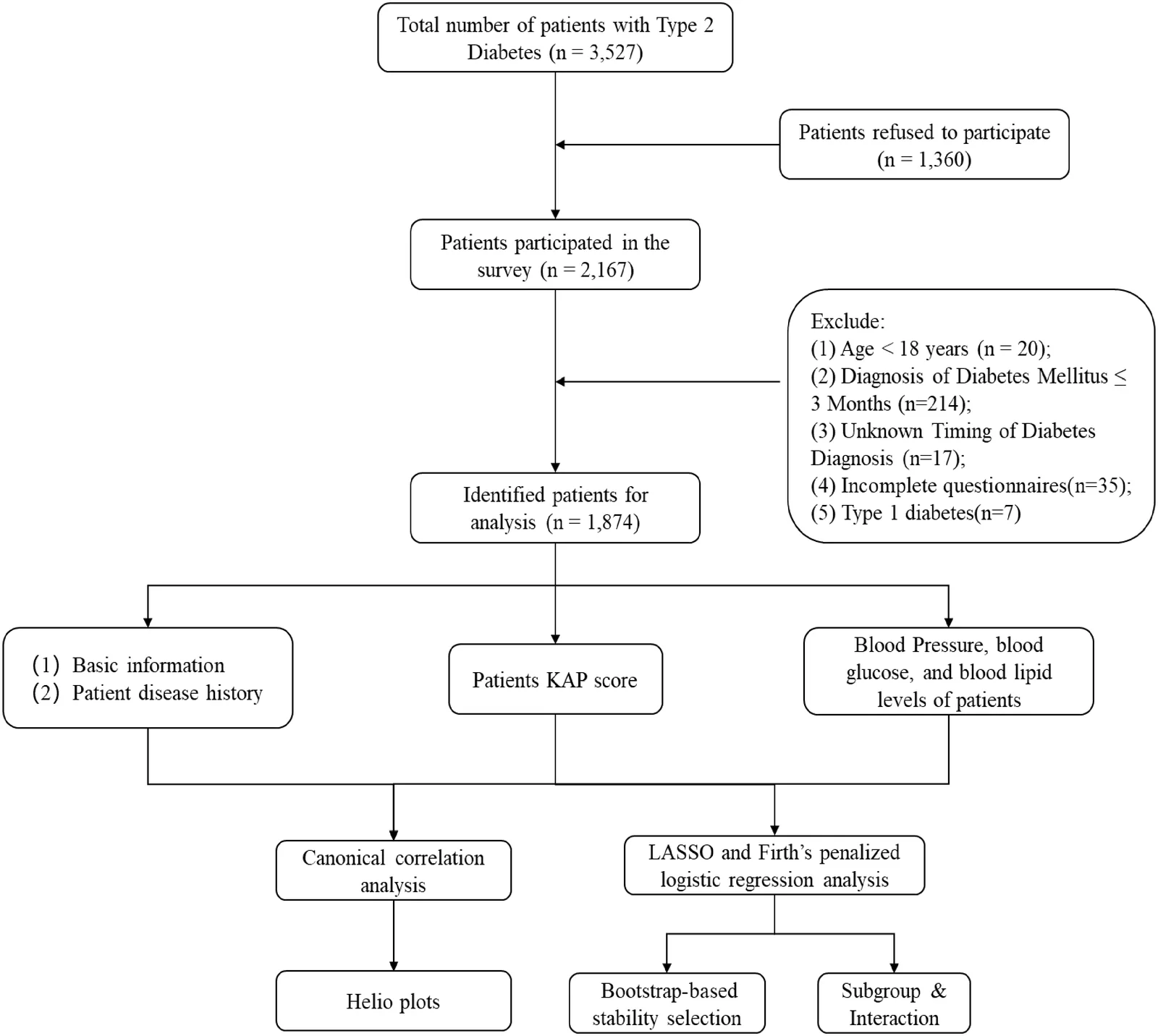
Flowchart of data selection and analysis in patients with T2DM.

**Figure 2:**
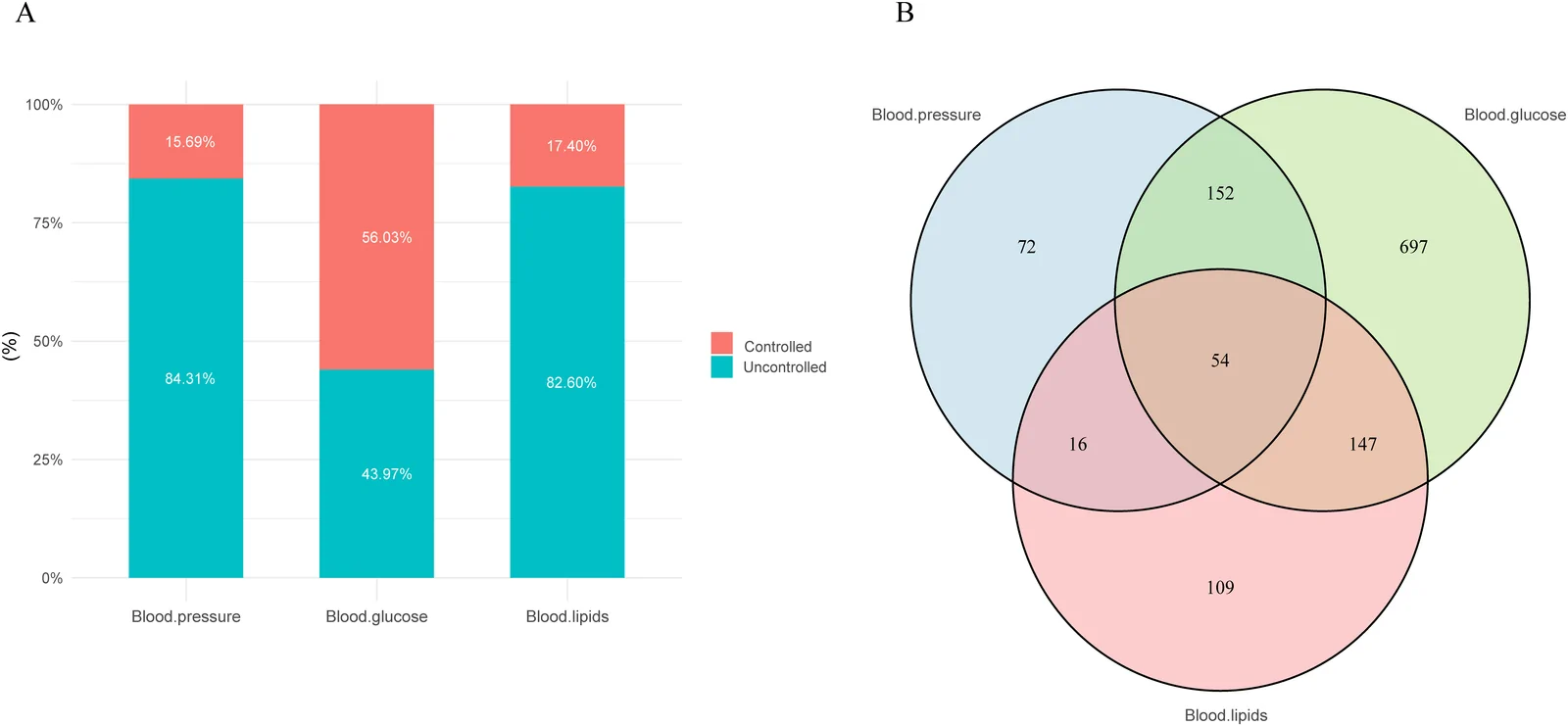
Compliance status of blood pressure, blood glucose, and blood lipids **(A)** and the combined target achievement **(B)** in patients with T2DM. **(A)** Stacked bar chart showing the achievement rates of the blood pressure, blood glucose, and blood lipids in patients with T2DM. Red indicates controlled status, and cyan indicates uncontrolled status. **(B)** Venn diagram illustrating the overlap of patients achieving blood pressure, blood glucose, and blood lipid targets simultaneously. Numbers in each section indicate the number of patients.

Of the initial 2,167 participants, 293 were excluded: 241 for not meeting the predefined eligibility criteria (age <18 years, diabetes duration ≤3 months, or type 1 diabetes), and 52 for data-quality or data-completeness issues. Consequently, 1,874 participants were included in the final analysis (**Figure 1**). Among these, 15.69% (n = 294) achieved the blood pressure target, 56.03% (n = 1,050) achieved the glycemic target, and 17.40% (n = 326) achieved the lipid target (**Figure 2A**). Only 54 participants (2.88%) achieved all three targets simultaneously (**Figure 2B**).

Participants had a mean age of 57.17 years, and 40.66% were female. Most participants resided in urban areas (63.55%). The most common monthly income range was 2,000–4,000 CNY (32.34%), and 87.94% were covered by basic medical insurance. The most frequent education level was middle school (30.36%), and 30.26% worked as farmers. In addition, 4.59% were divorced or single, and 24.71% were current smokers.

Regarding medical history, 29.99% (n=562) had FHDM; 45.78% (n=858) had diabetes duration ≥10 years; 41.62% (n=780) had HHTN; 4.80% (n=90) had stroke history; 11.37% (n=213) had CHD; 1.76% (n=33) had ACS; 2.45% (n=46) had PVDWS; 4.75% (n=89) had FHCHD; 2.29% (n=43) had FH; 3.20% (n=60) had CABG or PCI; and 2.35% (n = 44) had RI (**Table 2**).

**Table 2:** Baseline characteristics and disease history of patients with T2DM according to combined target achievement.

Characteristics	Total(N=1874)	Achieving the combined target goal(N=54)	Not achieving the combined target goal(N=1820)	χ2 or t	*P*
Age(years), mean ± SD	57.17±13.26	56.28±13.4	57.19±13.26	-0.49	0.62
Gender, N (%)					
Male	1112	29(53.7)	1083(59.51)	0.73	0.39
Female	762	25(46.3)	737(40.49)		
BMI, N (%)				22.58	0.00
<18.5	249	18(33.33)	231(12.69)		
18.5≤, <24	1207	32(59.26)	1175(64.56)		
≥24	418	4(7.41)	414(22.75)		
Monthly income (CNY), N (%)				4.50	0.34
Below 1000	389	14(25.93)	375(20.6)		
1000-2000	432	11(20.37)	421(23.13)		
2000-4000	606	14(25.93)	592(32.53)		
4000-6000	340	9(16.67)	331(18.19)		
Above 6000	107	6(11.11)	101(5.55)		
Place of residence, N (%)				0.59	0.44
Urban areas	1191	37(68.52)	1154(63.41)		
Rural areas	683	17(31.48)	666(36.59)		
Health insurance coverage, N (%)				7.17	0.11
Basic social medical insurance	1648	44(81.48)	1604(88.13)		
Commercial insurance	10	1(1.85)	9(0.49)		
Out-of-pocket medical care	71	1(1.85)	70(3.85)		
Publicly-funded medical care	112	7(12.96)	105(5.77)		
Basic social medical insurance combine with Commercial insurance	33	1(1.85)	32(1.76)		
Education level, N (%)				25.09	0.00
Graduate student	7	2(3.7)	5(0.27)		
Bachelor	283	12(22.22)	271(14.89)		
Junior college	295	13(24.07)	282(15.49)		
Technical secondary or high school	506	14(25.93)	492(27.03)		
Middle school	569	10(18.52)	559(30.71)		
Primary school	214	3(5.56)	211(11.59)		
Working conditions, N (%)				8.78	0.12
Currently employed	540	20(37.04)	520(28.57)		
Retired	524	13(24.07)	511(28.08)		
farmer	567	11(20.37)	556(30.55)		
Individual traders	142	8(14.81)	134(7.36)		
Unemployed or jobless	85	1(1.85)	84(4.62)		
Student	16	1(1.85)	15(0.82)		
Marital status, N (%)				0.24	0.90
Single	55	1(1.85)	54(2.97)		
Divorced	31	1(1.85)	30(1.65)		
Married or cohabiting	1788	52(96.3)	1736(95.38)		
Smoking status, N (%)				1.53	0.47
Never smoke	1109	36(66.67)	1073(58.96)		
Former smokers, who have quit smoking	302	6(11.11)	296(16.26)		
Current smokers	463	12(22.22)	451(24.78)		
FHDM, N (%)				1.60	0.21
No/Unknown	1312	42(77.78)	1270(69.78)		
Yes	562	12(22.22)	550(30.22)		
T2DM.duration, year				2.74	0.38
>0.25,<1	15	1(1.85)	14(0.77)		
≥1,<3	387	15(27.78)	372(20.44)		
≥3,<10	614	17(31.48)	597(32.8)		
≥10	858	21(38.89)	837(45.99)		
HHTN, N (%)				5.64	0.02
No/Unknown	1094	40(74.07)	1054(57.91)		
Yes	780	14(25.93)	766(42.09)		
Stroke history, N (%)				1.06	0.52
No/Unknown	1784	53(98.15)	1731(95.11)		
Yes	90	1(1.85)	89(4.89)		
CHD, N (%)				0.86	0.35
No/Unknown	1661	50(92.59)	1611(88.52)		
Yes	213	4(7.41)	209(11.48)		
ACS, N (%)				5.20	0.06
No/Unknown	1841	51(94.44)	1792(98.46)		
Yes	33	3(5.56)	28(1.54)		
PVDWS, N (%)				0.08	1.00
No/Unknown	1828	53(98.15)	1775(97.53)		
Yes	46	1(1.85)	45(2.47)		
FHCHD, N (%)				0.87	0.32
No/Unknown	1785	50(92.59)	1735(95.33)		
Yes	89	4(7.41)	85(4.67)		
FH, N (%)				0.05	1.00
No/Unknown	1831	53(98.15)	1778(97.69)		
Yes	43	1(1.85)	42(2.31)		
CABG or PCI, N (%)				0.05	0.69
No/Unknown	1814	52(96.3)	1762(96.81)		
Yes	60	2(3.7)	58(3.19)		
RI, N (%)				0.45	0.36
No/Unknown	1830	52(96.3)	1778(97.69)		
Yes	44	2(3.7)	42(2.31)		

BMI, body mass index; FHDM, family history of diabetes mellitus; T2DM duration, duration of type 2 diabetes mellitus; HHTN, history of hypertension; Stroke history, history of stroke; CHD, history of coronary heart disease; ACS, acute coronary syndrome within the past year; PVDWS, symptomatic peripheral vascular disease; FHCHD, family history of premature coronary heart disease; FH, familial hypercholesterolemia; CABG or PCI, prior coronary artery bypass grafting or percutaneous coronary intervention; RI, renal insufficiency.

In univariate analyses, lower education level, higher BMI, and presence of HHTN were each associated with an increased likelihood of poor cardiometabolic control (*P*<0.05).

### 3.2 KAP scores of participants

The mean scores for knowledge, attitude, and practice were 61.35±22.95, 79.13±16.90, and 75.95±14.86, respectively. Among the knowledge subscales, the score for diabetes-related knowledge was highest, whereas the score for lipid-related knowledge was lowest. For the practice domain, self-care management scores were highest, whereas self-care monitoring scores were lowest (**Table 3**).

**Table 3:** KAP scores among patients with T2DM stratified by achievement of combined target goals.

Characteristics	Variable	Total(N=1874)	Achieving the combined target goal(N=54)	Not achieving the combined target goal(N=1820)	*t*	*P*
Knowledge	Diabetes	74.46±30.92	79.26±25.17	74.32±31.06	1.41	0.16
	Hypertension	54.04±25.80	60.74±13.99	53.85±26.04	3.45	0.00
	Hyperlipidemia	52.71±30.74	62.59±21.99	52.42±30.92	3.30	0.00
	Medication	57.95±33.97	65.74±31.49	57.72±34.02	1.84	0.07
	General	67.59±28.42	73.33±23.95	67.42±28.53	1.78	0.08
	Total score	61.35±22.95	68.33±14.71	61.14±23.12	3.47	0.00
Attitude		79.13±16.90	80.69±19.48	79.08±16.82	0.60	0.55
Practices	Self-care maintenance	74.36±15.14	79.01±15.12	74.23±15.12	2.29	0.03
	Self-care monitoring	72.16±16.70	78.06±15.53	71.98±16.71	2.83	0.01
	Self-care management	79.13±18.47	85.72±17.05	78.94±18.48	2.87	0.01
	Self-care confidence	78.13±16.34	83.43±17.01	77.97±16.29	2.33	0.02
	Total score	75.95±14.86	81.56±13.82	75.78±14.86	3.02	0.00

### 3.3 Canonical correlation analysis

CCA was performed to examine the multivariate relationships between demographic characteristics (X1), medical history (X2), and KAP scores (X3) as independent variable sets, and the three components of cardiometabolic control—attainment of blood pressure, glycemic, and lipid targets (Y)—as the dependent variable set. All three canonical correlations were statistically significant (*P*<0.05). The first canonical correlation coefficients for X1–Y, X2–Y, and X3–Y were 0.28, 0.38, and 0.13, respectively, indicating weak-to-moderate shared variance between each independent variable set and the pattern of cardiometabolic control (**Table 4**).

**Table 4:** Canonical correlation analysis of variables.

Variables	Canonical correlation coeffcient	F	*p*
X1*vs* Y	0.28	8.10	0.00
X2*vs* Y	0.38	10.59	0.00
X3*vs* Y	0.13	4.57	0.00

The F statistic is derived from Wilks' Lambda and is used to test the statistical significance of each canonical function.

**Figure 3:**
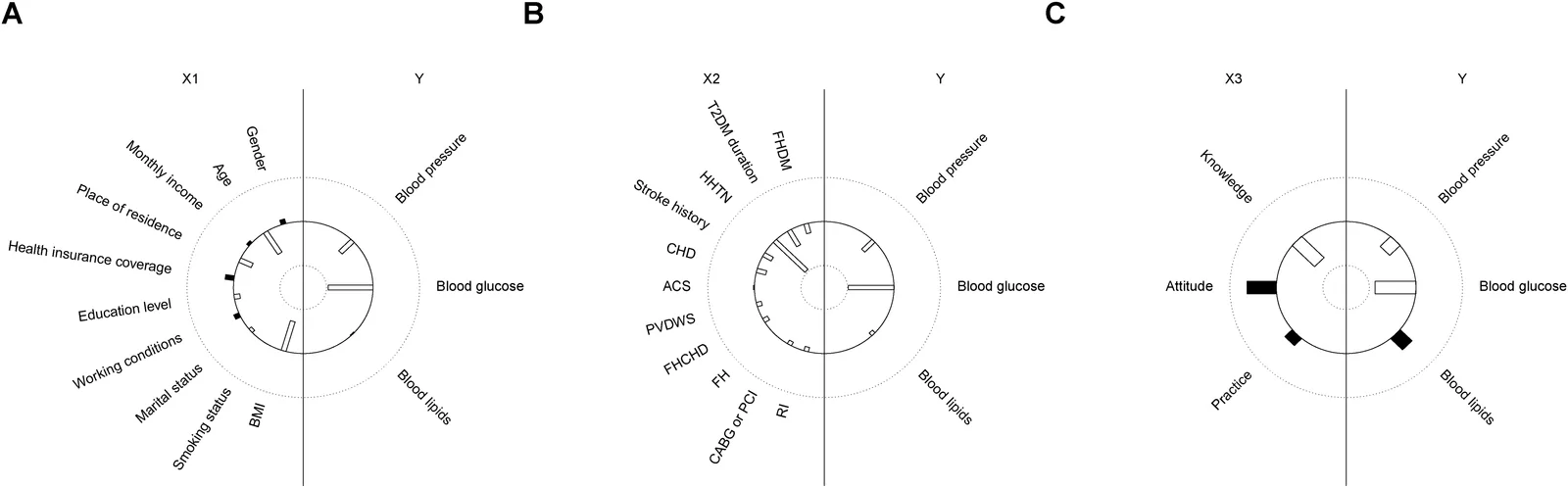
Canonical correlation analysis of patient information and the cardiometabolic indexes. **(A)** Canonical correlation helio plot showing structural correlations between patients’ demographic characteristics (X1) and cardiometabolic control (Y). **(B)** Canonical correlation helio plot showing structural correlations between patients’ medical history (X2) and cardiometabolic control (Y). **(C)** Canonical correlation helio plot showing structural correlations between patients’ KAP score (X3) and cardiometabolic control (Y). The solid black bars indicate positive correlations, and the hollow white bars indicate negative correlations. The length of the bars represents the magnitude of the structure coefficients. BMI, body mass index; FHDM, family history of diabetes mellitus; T2DM duration, duration of T2DM; HHTN, history of hypertension; Stroke history, history of stroke; CHD, history of coronary heart disease; ACS, acute coronary syndrome within the past year; PVDWS, symptomatic peripheral vascular disease; FHCHD, family history of premature coronary heart disease; FH, familial hypercholesterolemia; CABG or PCI, prior coronary artery bypass grafting or percutaneous coronary intervention; RI, renal insufficiency; KAP, knowledge-attitude-practice.

Structure coefficients from the three canonical functions are shown in the helio plots (**Figure 3**). Across all three canonical functions, blood glucose consistently showed the highest structure coefficient among the Y-side components (range: -0.88 to -0.98), whereas blood lipids contributed minimally and blood pressure showed intermediate loadings, indicating that the canonical relationships predominantly reflected associations with glycemic control rather than with blood pressure or lipid control.

Among the independent variables, BMI (r = -0.70) and age (r = -0.57) showed the largest contributions within the demographic characteristics set (X1); HHTN (r = -0.96) showed a markedly larger contribution than the other variables within the medical history set (X2), including T2DM duration (r = -0.40); and knowledge (r = -0.68) and attitude (r = 0.63) showed the largest, oppositely signed contributions within the KAP score set (X3), while practice contributed comparatively less (r = 0.22).

### 3.4 Features selected in Lasso−Firth regression model

**Figure 4:**
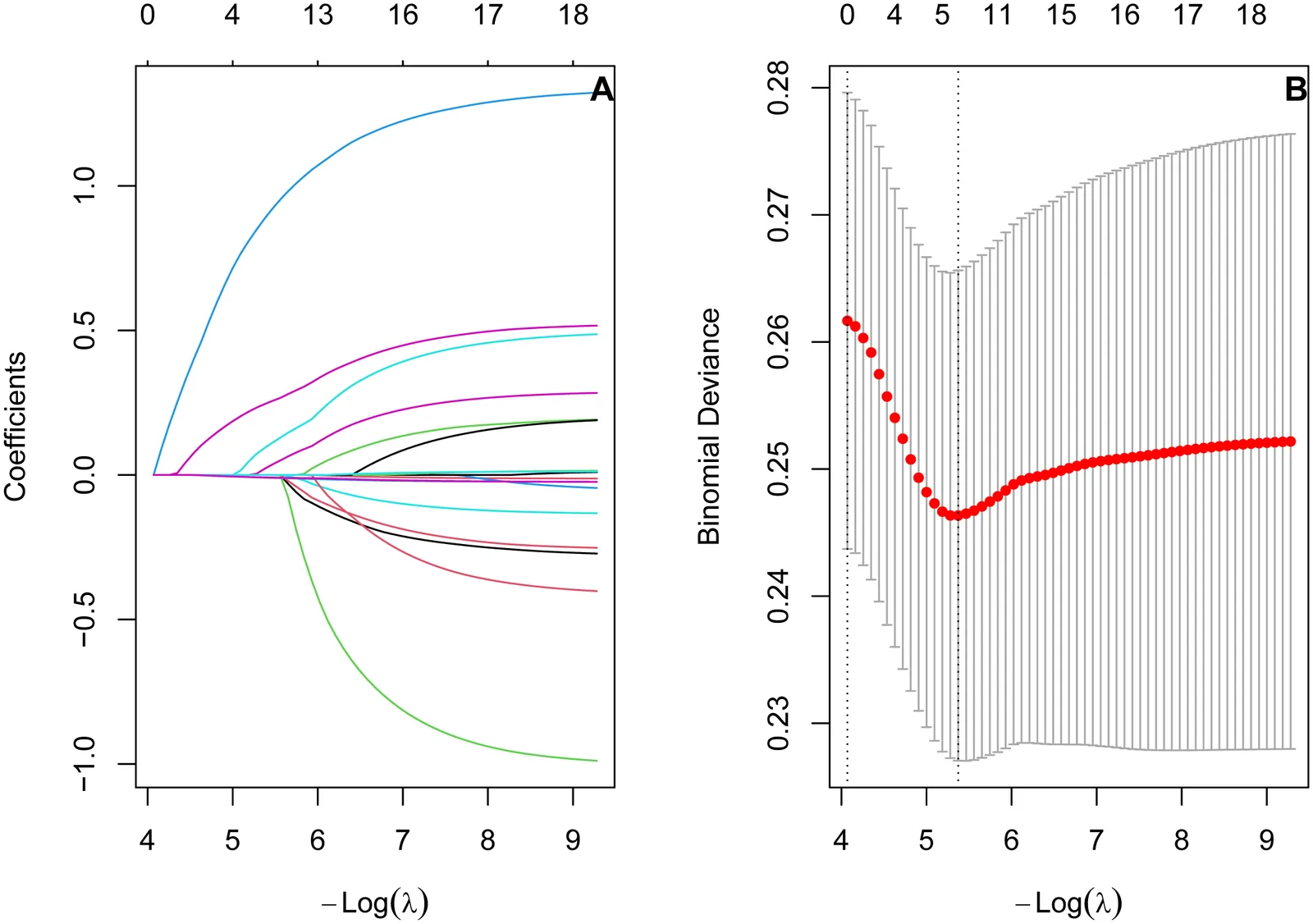
Variable screening using LASSO regression. **(A)** LASSO regression coefficient path plot. **(B)** LASSO regression cross-validation plot.

LASSO regression was used to perform variable selection and coefficient shrinkage, thereby minimizing the risk of overfitting (**Figure 4**). All candidate variables demonstrated VIF values below 2 (maximum VIF=1.89), indicating the absence of substantial multicollinearity (**Supplementary Table 1**); similarly, the six variables retained in the final model showed very low VIF values (maximum VIF=1.23). Selection frequencies for all candidate variables were also examined (**Supplementary Table 2**), and the six variables retained in the final model all demonstrated high selection stability (>75%). The model retained 6 of 18 candidate variables associated with poor cardiometabolic control: education level, BMI, T2DM duration, HHTN, knowledge score, and practice score. Of these variables, BMI, education level, and knowledge score demonstrated the strongest and most consistent associations with poor cardiometabolic control (**Table 5**). These factors exhibited high selection stability (≥81% across 1,000 resamples) and maintained nominally significant associations in the Firth regression model (*P*<0.05), which remained robust across varying selection thresholds (60%–80%; **Supplementary Table 3**).

**Table 5:** Variable selection using multivariable LASSO-Firth regression analysis.

Characteristic	Multivariate analysis		
	OR	CI	*P*
Education. Level			
Graduate student	Reference		
Bachelor	10.46	1.51-59.45	0.020
Junior college	10.65	1.57-59.65	0.019
Technical secondary or high school	17.88	2.64-99.07	0.005
Middle school	28.52	4.11-164.36	0.002
Primary school	26.06	3.23-196.83	0.004
BMI			
<18.5	Reference		
18.5-23.9	3.87	1.98-7.41	<0.001
>=24	11.53	3.92-42.40	<0.001
T2DM.duration			
>0.25,<1	Reference		
≥1,<3	2.53	0.20-15.11	0.411
≥3,<10	3.33	0.27-19.75	0.295
≥10	3.66	0.30-21.81	0.263
HHTN			
No/Unknown	Reference		
Yes	1.31	0.69-2.58	0.414
Knowledge	0.98	0.96-1.00	0.009
Practices	0.99	0.96-1.01	0.195

Variables selected via bootstrap-based stability selection, 1,000 resamples; primary threshold: selection frequency ≥ 75%. BMI, body mass index; T2DM duration, duration of type 2 diabetes mellitus; HHTN, history of hypertension.

### 3.5 The sensitivity analysis of knowledge

Knowledge score was further categorized into quartiles for a sensitivity analysis. Compared with the lowest quartile (Q1), Q2 showed no statistically significant difference in risk (OR=0.43, *P*=0.073). Although Q3 and Q4 showed a significantly decreased risk of poor cardiometabolic control compared with Q1 (both *P*<0.05), the overall dose–response trend did not reach the pre-specified significance threshold (*P* for trend = 0.06). This finding should therefore be interpreted with caution (**Table 6**).

**Table 6:** Sensitivity analysis of the effect of knowledge score quartiles on the risk of poor cardiometabolic control.

Knowledge Quartile	Scores	OR	95% CI	P value	P for trend
Q1	<51	Reference			0.061
Q2	≥51,<66.5	0.43	0.16-1.08	0.073	
Q3	≥66.5,<77.5	0.36	0.13-0.90	0.028	
Q4	≥77.5	0.36	0.12-0.99	0.048	

Adjusted for education level, BMI, T2DM duration, history of hypertension, and practices score using Firth’s penalized logistic regression. Q1 represents the lowest quartile (reference), and Q4 represents the highest quartile.

### 3.6 The subgroup analysis of knowledge

**Figure 5:**
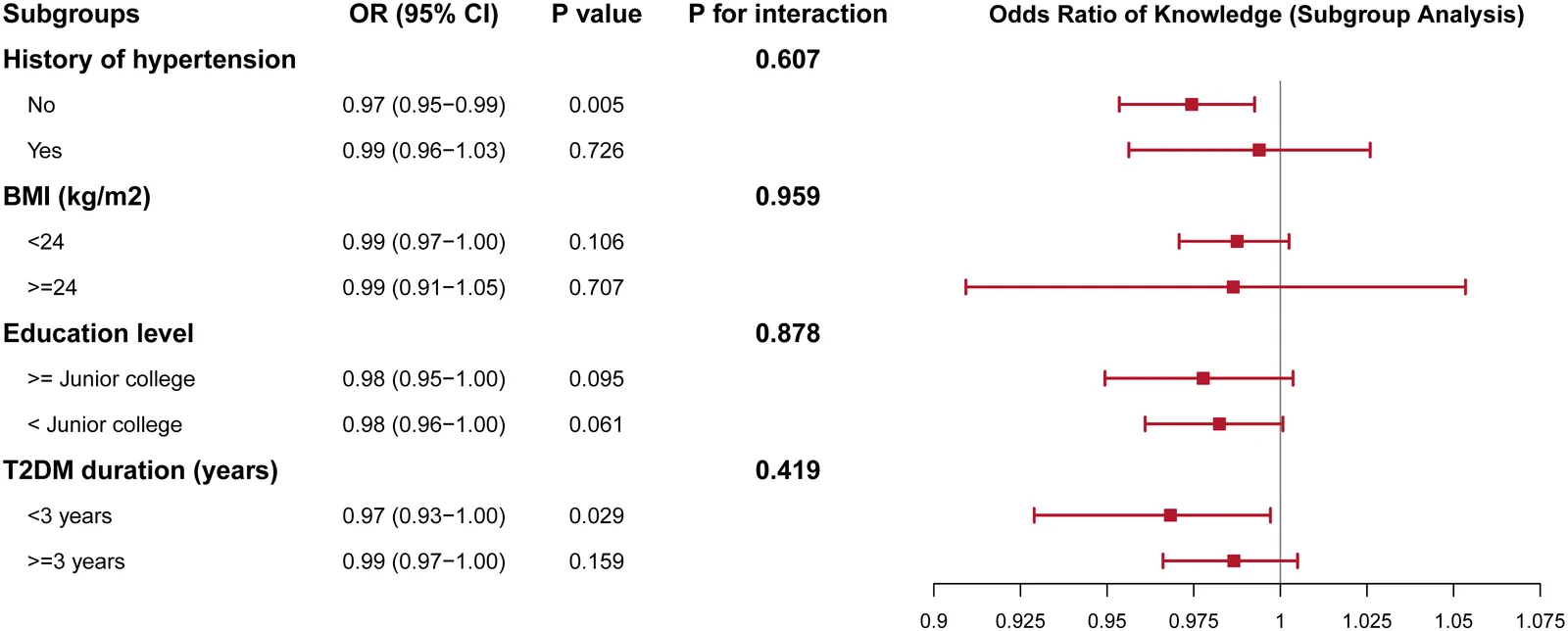
Forest plot of subgroup analyses and tests for interaction. BMI, body mass index; T2DM duration, duration of type 2 diabetes mellitus.

Subgroup analyses were conducted to assess whether the association between knowledge score and poor cardiometabolic control varied across subpopulations (**Figure 5**). No significant interactions were found for education level, BMI, T2DM duration, or HHTN (all *P* for interaction > 0.05).

## 4 Discussion

This cross-sectional study found that the overall rate of comprehensive cardiometabolic control among patients with T2DM in Shanxi Province was only 2.88%, which is comparable to the rate reported among patients in Shanghai between 2014 and 2016 (12). Regarding individual cardiometabolic components, 56.03% of patients achieved the glycemic target, which is consistent with findings from previous national multicenter studies (9, 25–29), suggesting that approximately half of the patients achieved adequate glycemic control. In contrast, only 15.69% of patients achieved target blood pressure control, which was lower than the national average (9). Lipid targets were achieved by 17.40% of patients, slightly exceeding the proportion reported in the Shanghai cohort. Taken together, these findings indicate that blood pressure and lipid control remain suboptimal among patients with T2DM in Shanxi Province and highlight the need for more effective management strategies to improve these cardiometabolic indicators and potentially reduce the burden of diabetes-related complications.

The KAP model provides a theoretical framework linking health knowledge, attitude, and self-care behaviors to cardiometabolic management. In this study, participants who achieved comprehensive cardiometabolic control demonstrated significantly higher knowledge and practice scores than those who did not, whereas attitude scores did not differ significantly between the two groups. This suggests that diabetes-related knowledge and self-management behaviors—rather than attitude alone—may be more closely linked to cardiometabolic control in this population, although causal direction cannot be established from the current cross-sectional design. Notably, relatively low knowledge scores related to blood pressure management, lipid control, and medication use were observed, indicating specific educational gaps that may contribute to suboptimal control in these domains and representing actionable targets for future diabetes education programs. This knowledge gap may also extend to the practice dimension: the comparatively low self-monitoring scores observed in this sample may reflect limited patient ability to actively track and recognize relevant clinical signs, thereby reducing opportunities for timely self-adjustment or medical consultation. Taken together, these findings suggest shortcomings in practical skills training and scenario-based education, whereby knowledge is not always effectively translated into actionable daily behaviors. Within the practice dimension of the KAP model, dietary behavior constitutes a key, modifiable component of diabetes self-management and is closely linked to glycemic and lipid control. Emerging evidence on functional foods and food–medicine homologous resources illustrates the broader range of dietary strategies that could inform future, more comprehensive diabetes education programs (30–33), although these specific components were not evaluated in the present study.

The CCA provided complementary insight into these associations. Notably, the canonical relationships were driven predominantly by glycemic control rather than by blood pressure or lipid control, and knowledge—rather than attitude—emerged as the KAP component most consistently linked to better glycemic control, corroborating the bootstrap stability selection and Firth regression findings described below, in which knowledge, but not attitude, was retained as a stably selected variable associated with poor cardiometabolic control. HHTN also showed an unexpectedly large contribution, driven primarily by its association with glycemic control rather than blood pressure—a pattern whose underlying mechanisms warrant further investigation.

Using a bootstrap stability selection framework followed by Firth’s penalized logistic regression, this study identified BMI, education level, and knowledge as the variables most consistently and significantly associated with poor cardiometabolic control in this population. Practice score, HHTN, and T2DM duration were also stably selected but did not reach nominal statistical significance, suggesting that these variables may represent weaker or more context-dependent associations warranting confirmation in future studies. Given the post-selection nature of the reported inferential statistics, the findings should be interpreted as exploratory and hypothesis-generating; independent external validation with a pre-specified analytical plan is needed before these associations can be considered confirmed. Together with the CCA findings described above, these two complementary multivariate approaches converge on knowledge, rather than attitude, as the KAP component most robustly linked to cardiometabolic—particularly glycemic—control, and further illustrate that variable importance can differ across analytical frameworks depending on how the outcome is operationalized.

To further characterize the extent to which our composite-outcome findings reflect domain-specific patterns, we applied the same bootstrap stability selection and Firth regression pipeline separately to each of the three individual domains (blood pressure, glycemic, and lipid control) as binary outcomes (**Supplementary Table 4**). BMI was significantly associated with poor control across all three domains, reinforcing its status as the most robust finding in this study. Education level was significantly associated with poor blood pressure and lipid control but not glycemic control, whereas practice score and HHTN were significantly associated with poor glycemic control (and, for practice score, lipid control) but not blood pressure control. Notably, HHTN’s domain-specific pattern—significant for glycemic but not blood pressure control—corroborates the CCA findings above, in which HHTN’s contribution was likewise driven primarily by its association with glycemic control, strengthening confidence in this pattern across independent analyses. Several variables absent from the primary six-variable model—including FHCVD, age, place of residence, health insurance coverage, smoking status, gender, and monthly income—emerged as significant in one or more domain-specific models, indicating that the stringent, all-or-none composite definition may obscure domain-specific associated factors. Conversely, knowledge score, significant in the composite analysis, reached significance in no single domain, suggesting a diffuse, cross-domain association—small, sub-threshold associations across domains that become detectable only when aggregated—rather than a strong domain-specific association. These findings should inform interpretation of the composite-outcome results, including those related to knowledge, and reinforce the exploratory framing adopted throughout this study. The observed pattern—in which higher knowledge quartiles were associated with reduced risk, but the overall trend did not reach statistical significance—should be regarded as a hypothesis-generating observation rather than confirmatory evidence of a dose–response relationship. This finding may suggest that a protective association with knowledge becomes apparent only beyond a certain threshold; however, this interpretation requires confirmation in future studies with larger samples or more granular exposure measures.

Diabetes has become a major public health burden owing to its high prevalence, complex complications, and frequent coexistence with hypertension and dyslipidemia. However, most previous studies have focused primarily on glycemic control, with relatively few simultaneously evaluating blood pressure and lipid management or integrating real-world clinical and behavioral data to comprehensively assess cardiometabolic risk in patients with T2DM. To address this gap, this multicenter cross-sectional study was conducted across seven medical institutions in Shanxi Province, systematically collecting patients’ clinical characteristics and KAP information, and identifying candidate variables associated with poor cardiometabolic control using CCA, bootstrap stability selection, and Firth penalized regression analyses. This study leverages not only a large multicenter sample and routine clinical testing data but also patients’ KAP information obtained from questionnaire surveys, thereby enhancing the practical relevance of the analysis.

This study has several limitations. First, the study population was primarily drawn from outpatients and inpatients receiving care in the endocrinology departments of provincial and municipal tertiary hospitals. Compared with patients managed in primary care or community settings, these patients may differ systematically in disease severity, health-seeking behavior, and access to self-management resources, potentially limiting the generalizability of our findings to the broader population of patients with T2DM in the region. In addition, of the 3,527 eligible patients approached, 1,360 (38.6%) declined participation. Patients who agreed to complete the KAP survey and undergo clinical data linkage may differ systematically from those who declined—for example, in health literacy, engagement with self-management, or disease severity. We were unable to obtain demographic or clinical information for non-participants, owing to ethical and practical constraints on data collection for individuals who did not consent to participate; a formal comparison between participants and non-participants was therefore not feasible. The direction and magnitude of any resulting selection bias cannot be determined, and this represents an additional source of uncertainty regarding the generalizability of our findings. Further, “unknown” responses regarding specialized cardiovascular medical histories were conservatively coded as absence of a confirmed diagnosis (“no/unconfirmed”). Although this approach is consistent with common practice in clinical epidemiological surveys, it may have underestimated the prevalence of conditions requiring specialized diagnostic evaluation. Second, although we employed bootstrap-based stability selection to improve the robustness of variable selection before Firth penalized logistic regression, the reported odds ratios, 95% confidence intervals, and P values were derived from a model fitted to the same dataset used for variable selection. Consequently, these estimates do not fully account for the uncertainty introduced during the variable-selection process and should not be interpreted as valid confirmatory inferential statistics; instead, they should be regarded as exploratory and hypothesis-generating. Although sensitivity analyses using alternative stability-selection thresholds (60–80%) demonstrated that the core associations (BMI, education level, and knowledge score) were robust to this methodological choice, confirmation in independent external cohorts using pre-specified analytical protocols is required before these associations can be considered established. Third, this study employed a cross-sectional design and therefore can identify associations but cannot establish temporal or causal relationships between cardiometabolic control and KAP-related variables. Prospective cohort studies and intervention trials are needed to validate these findings and clarify their temporal and causal relationships.

Despite these limitations, the findings may inform future diabetes management strategies. Educational interventions may benefit from placing greater emphasis on knowledge related to hypertension, dyslipidemia, and medication use, while strengthening self-management skills and symptom recognition through structured education and follow-up programs. Future studies should evaluate whether interventions such as home-based monitoring, community education, practical skills training, and simulation-based education can improve cardiometabolic control in patients with T2DM.

## 5 Conclusions

Based on the questionnaire and clinical data collected in this study, BMI, education level, and knowledge score emerged as the variables most consistently associated with poor cardiometabolic control in patients with T2DM, whereas practice score, hypertension history, and T2DM duration represent additional candidate variables warranting further investigation. Given the cross-sectional and exploratory nature of this study, these findings should be interpreted as hypothesis-generating rather than confirmatory. Future studies should validate these associations in independent cohorts, particularly in primary care and rural populations, and clarify their temporal and causal relationships through prospective cohort studies and intervention trials.

## Data Availability

The raw data supporting the conclusions of this article will be made available by the authors, without undue reservation.
