# Supplementary material for: Factors associated with poor cardiometabolic control in patients with type 2 diabetes: a Lasso-Firth penalized regression analysis
Source: Front Endocrinol (Lausanne). 2026 Sep 3;17:1905392. doi: 10.3389/fendo.2026.1905392 (PMC13581911; doi:10.3389/fendo.2026.1905392)
Supplement: Supplementary file 1 [file Table1.docx]

Table S1 VIFs before and after variable selection

| **Variables** | **Pre-selection VIF** | **Post-selection VIF** |
| --- | --- | --- |
| Gender | 1.72 | — |
| Age | 1.64 | — |
| Monthly income | 1.89 | — |
| Place of residence | 1.24 | — |
| Health insurance coverage | 1.27 | — |
| Education level | 1.48 | 1.05 |
| Working conditions | 1.69 | — |
| Marital status | 1.20 | — |
| Smoking status | 1.59 | — |
| BMI | 1.29 | 1.18 |
| HHTN | 1.23 | 1.10 |
| T2DM duration | 1.27 | 1.05 |
| FHCVD | 1.20 | — |
| ASCVD | 1.22 | — |
| RI | 1.04 | — |
| Knowledge | 1.32 | 1.23 |
| Attitude | 1.69 | — |
| Practice | 1.79 | 1.18 |

Note: A VIF value < 5 indicates the absence of substantial multicollinearity. Initial model includes all 18 candidate variables; final model includes the 6 variables retained after bootstrap-based stability selection.
