# Supplementary material for: Factors associated with poor cardiometabolic control in patients with type 2 diabetes: a Lasso-Firth penalized regression analysis
Source: Front Endocrinol (Lausanne). 2026 Sep 3;17:1905392. doi: 10.3389/fendo.2026.1905392 (PMC13581911; doi:10.3389/fendo.2026.1905392)
Supplement: Supplementary file 2 [file Table2.docx]

Table S2 Selection Frequency of Candidate Variables Based on 1000 Bootstrap LASSO Resamples

| **Variable** | **Selection Frequency (%)** | **Variable** | **Selection Frequency (%)** |
| --- | --- | --- | --- |
| BMI | 99.9 | Monthly income | 64.5 |
| Education level | 99.1 | Gender | 61.4 |
| Knowledge | 99.1 | ASCVD | 60.9 |
| Practice | 87.8 | FHCAD | 59.1 |
| HHTN | 81.5 | Age | 58.6 |
| T2DM duration | 78.5 | Place of residence | 55.3 |
| Marital status | 74.4 | Smoking status | 54.3 |
| Health insurance coverage | 72 | Attitude | 54.1 |
| RI | 69.5 | Working conditions | 50.5 |
