# Supplementary material for: Factors associated with poor cardiometabolic control in patients with type 2 diabetes: a Lasso-Firth penalized regression analysis
Source: Front Endocrinol (Lausanne). 2026 Sep 3;17:1905392. doi: 10.3389/fendo.2026.1905392 (PMC13581911; doi:10.3389/fendo.2026.1905392)
Supplement: Supplementary file 3 [file Table3.docx]

Table S3. Sensitivity analysis of Firth regression estimates across alternative stability-selection thresholds (60–80%)

| **Threshold Model** | **Variables** | **OR (95% CI)** | ***P*** |
| --- | --- | --- | --- |
| Model_1 (>=80%) | BMI | 3.571 (2.089-6.193) | <0.001 |
| Model_1 (>=80%) | Education level | 1.481 (1.185-1.861) | <0.001 |
| Model_1 (>=80%) | Knowledge | 0.981 (0.964-0.996) | 0.014 |
| Model_1 (>=80%) | Practices | 0.985 (0.962-1.007) | 0.189 |
| Model_1 (>=80%) | HHTN | 1.453 (0.786-2.819) | 0.238 |
| Model_2 (>=75%) | BMI | 3.595 (2.113-6.207) | <0.001 |
| Model_2 (>=75%) | Education level | 1.457 (1.163-1.834) | 0.001 |
| Model_2 (>=75%) | Knowledge | 0.981 (0.964-0.996) | 0.012 |
| Model_2 (>=75%) | Practices | 0.985 (0.962-1.007) | 0.181 |
| Model_2 (>=75%) | HHTN | 1.364 (0.730-2.670) | 0.337 |
| Model_2 (>=75%) | T2DM duration | 1.213 (0.860-1.702) | 0.267 |
| Model_3 (>=70%) | BMI | 3.676 (2.150-6.397) | <0.001 |
| Model_3 (>=70%) | Education level | 1.481 (1.177-1.871) | <0.001 |
| Model_3 (>=70%) | Knowledge | 0.980 (0.963-0.996) | 0.010 |
| Model_3 (>=70%) | Practices | 0.986 (0.963-1.008) | 0.225 |
| Model_3 (>=70%) | HHTN | 1.380 (0.735-2.709) | 0.322 |
| Model_3 (>=70%) | T2DM duration | 1.233 (0.875-1.728) | 0.229 |
| Model_3 (>=70%) | Marital status | 0.827 (0.509-1.154) | 0.295 |
| Model_3 (>=70%) | Health insurance coverage | 0.929 (0.733-1.217) | 0.572 |
| Model_4 (>=60%) | BMI | 3.602 (2.094-6.321) | <0.001 |
| Model_4 (>=60%) | Education level | 1.616 (1.268-2.067) | <0.001 |
| Model_4 (>=60%) | Knowledge | 0.978 (0.960-0.993) | 0.005 |
| Model_4 (>=60%) | Practices | 0.986 (0.962-1.009) | 0.232 |
| Model_4 (>=60%) | HHTN | 1.525 (0.798-3.043) | 0.205 |
| Model_4 (>=60%) | T2DM duration | 1.252 (0.886-1.761) | 0.200 |
| Model_4 (>=60%) | Marital status | 0.778 (0.476-1.099) | 0.171 |
| Model_4 (>=60%) | Health insurance coverage | 0.848 (0.656-1.129) | 0.246 |
| Model_4 (>=60%) | RI | 0.296 (0.087-1.542) | 0.130 |
| Model_4 (>=60%) | Monthly income | 1.234 (0.926-1.653) | 0.151 |
| Model_4 (>=60%) | Gender | 0.768 (0.425-1.399) | 0.386 |
| Model_4 (>=60%) | ASCVD | 0.611 (0.294-1.385) | 0.226 |
