# Supplementary material for: Factors associated with poor cardiometabolic control in patients with type 2 diabetes: a Lasso-Firth penalized regression analysis
Source: Front Endocrinol (Lausanne). 2026 Sep 3;17:1905392. doi: 10.3389/fendo.2026.1905392 (PMC13581911; doi:10.3389/fendo.2026.1905392)
Supplement: Supplementary file 4 [file Table4.docx]

**Table S4. Domain-specific Firth regression estimates for poor blood pressure, glycemic, and lipid control, following bootstrap-based stability selection**

| Outcome Measures | Variable | Selection Frequency (%) | OR (95% CI) | *P* |
| --- | --- | --- | --- | --- |
| Blood pressure | BMI | 100 | 1.598 (1.278 - 2.004) | 0.000 |
|  | T2DM duration | 100 | 1.478 (1.260 - 1.735) | 0.000 |
|  | Education level | 98.4 | 1.217 (1.087 - 1.364) | 0.001 |
|  | Health insurance coverage | 97.1 | 0.853 (0.757 - 0.964) | 0.012 |
|  | FHCVD | 94.5 | 1.355 (1.018 - 1.818) | 0.037 |
|  | Practices | 93.8 | 1.395 (0.940 - 2.132) | 0.101 |
|  | HHTN | 88.7 | 1.222 (0.921 - 1.628) | 0.166 |
|  | Place of residence | 84.4 | 0.772 (0.584 - 1.023) | 0.072 |
|  | Gender | 82.4 | 0.826 (0.596 - 1.141) | 0.247 |
|  | Smoking status | 79.3 | 1.106 (0.915 - 1.338) | 0.299 |
|  | Marital status | 77.2 | 0.890 (0.734 - 1.056) | 0.190 |
| Blood glucose | BMI | 100 | 1.683 (1.406 - 2.020) | 0.000 |
|  | HHTN | 100 | 3.689 (2.993 - 4.556) | 0.000 |
|  | Place of residence | 99.7 | 1.514 (1.212 - 1.893) | 0.000 |
|  | Age | 99.3 | 1.016 (1.006 - 1.025) | 0.001 |
|  | T2DM duration | 97.7 | 1.168 (1.015 - 1.344) | 0.030 |
|  | FHCVD | 94.5 | 1.268 (1.025 - 1.568) | 0.029 |
|  | Practices | 91.8 | 0.742 (0.553 - 0.993) | 0.045 |
|  | Monthly income | 87.1 | 1.143 (1.037 - 1.259) | 0.007 |
|  | Marital status | 79 | 0.878 (0.759 - 1.019) | 0.086 |
| Blood lipids | Education level | 99.7 | 1.278 (1.137 - 1.438) | 0.000 |
|  | BMI | 99.7 | 1.423 (1.140 - 1.782) | 0.002 |
|  | Age | 98.2 | 0.984 (0.973 - 0.995) | 0.005 |
|  | Place of residence | 96.2 | 0.711 (0.540 - 0.935) | 0.015 |
|  | Smoking status | 94.9 | 1.213 (1.017 - 1.450) | 0.032 |
|  | Practices | 94.6 | 0.601 (0.425 - 0.861) | 0.006 |
|  | Knowledge | 94.3 | 1.314 (0.995 - 1.745) | 0.054 |
|  | HHTN | 91.2 | 1.295 (0.991 - 1.698) | 0.059 |
|  | Gender | 86.2 | 1.372 (1.010 - 1.863) | 0.043 |
|  | T2DM duration | 84.1 | 0.914 (0.769 - 1.083) | 0.301 |
|  | Monthly income | 80.5 | 1.064 (0.932 - 1.216) | 0.360 |
|  | RI | 78.1 | 1.340 (0.583 - 3.747) | 0.516 |
|  | Marital status | 77.4 | 1.104 (0.924 - 1.303) | 0.267 |
|  | Working conditions | 75.1 | 0.936 (0.823 - 1.067) | 0.321 |
